# The bleeding costs of intensive vs. less intensive antithrombotic strategies: a meta-regression analysis

**DOI:** 10.1093/ehjqcco/qcaf128

**Published:** 2025-10-23

**Authors:** Sten E Deurvorst, Esther M E El-Hage – Verbeek, Nienke van Wingerden, Kirsten A Ziesemer, Jonne J Sikkens, Yvo M Smulders

**Affiliations:** Department of Internal Medicine, Amsterdam UMC, Meibergdreef 9, 1105 AZ Amsterdam, The Netherlands; Diabeter Rotterdam, Blaak 6, 3011 TA Rotterdam, The Netherlands; Department of Internal Medicine, Acibadem IMC, Arlandaweg 100, 1043 HP Amsterdam, The Netherlands; VU University, De Boelelaan 1105, 1081 HV Amsterdam, The Netherlands; Department of Internal Medicine, Amsterdam UMC, Meibergdreef 9, 1105 AZ Amsterdam, The Netherlands; Department of Internal Medicine, Amsterdam UMC, Meibergdreef 9, 1105 AZ Amsterdam, The Netherlands

**Keywords:** Antithrombotic therapy, Meta-regression analysis, Intracranial haemorrhage, Fatal haemorrhage, MACE

## Abstract

**Aims:**

Antithrombotic therapy is a cornerstone of secondary prevention of atherosclerotic cardiovascular disease (ASCVD). The main drawback of antithrombotic therapy is the associated bleeding risk. The correlation between the magnitude of antithrombotic efficacy and the severity of bleeding risk remains unclear, rendering the comparative evaluation of antithrombotic regimens based on their therapeutic advantages and risks challenging. This study aims to establish a benchmark for evaluating the trade-off between ASCVD prevention and bleeding risk through a systematic analysis of extant trial data.

**Methods and results:**

Meta-regression analysis. We searched PubMed and Embase and extracted trials comparing antithrombotic treatments in patients with established ASCVD. We extracted data on major adverse cardiovascular events (MACE) reduction and bleeding events. A weighted generalized additive regression was used for data analysis. Our analysis included 50 studies, comprising a total of 357 711 patients with coronary artery disease (63%), cerebrovascular events (28%), and peripheral arterial disease (15%). Overall, ASCVD event reduction by intensification of antithrombotic therapy correlates with an increased risk of intracranial haemorrhage. Also, the reduction in the composite of acute coronary syndrome and stroke is associated with an increased risk of fatal haemorrhage. When regimens not routinely used in current practice are excluded, the increased risk of intracranial haemorrhage with intensified antithrombotic treatment is attenuated to non-significant, but ASCVD event reduction still correlates with an increased risk in fatal haemorrhage. The analyses and figures we present can serve as a benchmark to compare results of future trials on antithrombotic regimens for ASCVD to other (contemporary) regimens.

**Conclusion:**

With currently used antithrombotic regimens, ASCVD event reduction by more intensive treatment no longer increases risk of intracranial bleeding. Fatal haemorrhage remains a seemingly inevitable cost of ASCVD reduction by antithrombotic treatment intensification, albeit at low absolute risks.

## Graphical Abstract

**Graphical Abstract:**
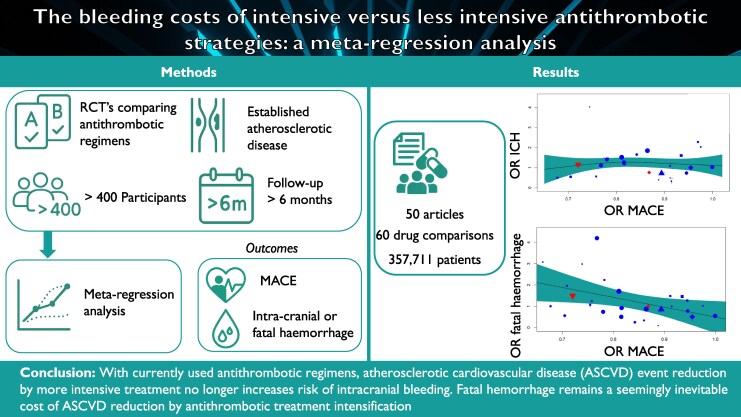

Key Learning Points What is already known:

- Intensified antithrombotic therapy reduces the risk of major adverse cardiovascular events (MACE) in patients with atherosclerotic cardiovascular disease.
- This benefit often comes with an increased risk of bleeding, particularly intracranial and fatal haemorrhage.
- There is limited quantitative data assessing the trade-off between cardiovascular benefit and bleeding risk across different antithrombotic regimens.

What this study adds:

- This meta-regression quantifies the empirical relationship between MACE reduction and bleeding risk across multiple trials.
- Currently used antithrombotic regimens no longer show a significant increase in intracranial haemorrhage, while a modest increase in fatal bleeding remains.
- The analysis provides a visual benchmark to compare benefit-risk profiles of future antithrombotic strategies.

## Introduction

Antithrombotic medication is the cornerstone of secondary prevention of atherosclerotic cardiovascular disease (ASCVD).^1^ The vast majority of trials show increased reduction of major adverse cardiovascular events (MACE) when using more intensive antithrombotic treatments. However, this benefit generally comes at the cost of increased bleeding risk. There is a lack of quantitative information about the trade-off between MACE reduction and bleeding increase. We therefore need to assess whether there is a common relation between the MACE reduction and bleeding increase which can then be used to guide the appraisal of current and future antithrombotic regimens. Regimens that compare favourably would be expected to provide more net benefit than average. We performed a meta-regression analysis of currently available trials comparing antithrombotic regimens enabling quantification of the empiric trade-off between antithrombotic benefit and bleeding risk.

## Methods

### Meta-regression protocol

A meta-regression analysis was conducted, with the protocol registered in the PROSPERO database (registration number CRD42023432104).

### Study inclusion criteria

The analysis focused on randomized clinical trials evaluating various antithrombotic strategies in patients with confirmed ASCVD. Exclusion criteria were as follows: trials with fewer than 400 participants per arm, treatment durations shorter than 6 months, inconsistent treatment durations across arms, and studies reporting results following an identical initial antithrombotic regimen. We excluded trials that compared antithrombotic drugs to placebo because this does not reflect current clinical practice in patients with established ASCVD.

### Outcome measures

Atherosclerotic cardiovascular disease was defined as coronary artery disease (CAD), stroke, and transient ischaemic attack (TIA) from a non-cardioembolic stroke, as well as peripheral arterial disease (PAD). Coronary artery disease could manifest as angina or an acute coronary syndrome (ACS). Amaurosis fugax was included as a subtype of TIA. For PAD, intermittent claudication and ischaemic foot disease were included.

### Study retrieval and selection

Literature searches were performed using PubMed and Embase, with the PubMed search strategy detailed in the Supplementary material. Merged results from both databases had duplicates removed. The selection process was assisted by ASReview LAB, which applies active machine learning to prioritize the most relevant references for appraisal.^2^ The first selection of references based on the title and abstract was performed by the first two authors. When 100 consecutive irrelevant references were excluded, the selection process was stopped. Full-text evaluation was conducted by the first three authors, with any discrepancies resolved by the last author. The review authors were not blinded to the journal titles, study authors, or institutions.

From the selected trials, we extracted the event leading to inclusion, antithrombotic treatments, the number of participants in each arm, incidence of composite and individual MACE components, incidence of specific and cumulative bleeding events, all-cause mortality, as well as cardiovascular mortality and mortality due to bleeding events. Outcomes of MACE included ischaemic stroke, ACS, revascularization, and major adverse limb events (MALE) (either revascularization or amputation). Bleeding events were subdivided in intracranial bleeding events, fatal bleeding events, hospital admission due to a bleeding event, significant haemoglobin drop, and the bleeding scale used (i.e. TIMI, BARC, ISTH). We planned to use a bleeding assessment tool as our primary bleeding outcome if one tool was used in 75% of trials or more. Because of the heterogeneity of the bleeding assessment tools, direct comparison between these tools was not possible. If the pre-specified condition was not met, we planned to use intracranial haemorrhage as the primary bleeding outcome because this is uniformly documented and it is the most clinically relevant bleeding complication.

### Data extraction and statistical analysis

Data extraction was independently performed by the first three authors. The efficacy of more vs. less intensive antithrombotic regimens was assessed using odds ratios derived from 2 × 2 contingency tables, applying the Haldane–Anscombe correction for zero-event instances.^3,4^ The antithrombotic regimen which was most efficacious in preventing the composite outcome of ACS and stroke was used as the numerator of the odds ratio.

We performed a meta-regression analysis to explore the relationship between cardiovascular outcomes and bleeding risk. We used a random effects generalized additive model (GAM) to allow for non-linearity of relations. Trials were weighted by their sample size. We pre-specified analyses to examine the influence of the event leading to inclusion and to perform the abovementioned analyses including only regimens used in contemporary practice. All analyses were performed using R Statistical Software (R version 4.2.1; R Core Team 2022). The GAM was performed using the mixed gam computation vehicle (mgcv) package.^5^

## Results

### Literature selection

**Figure 1:**
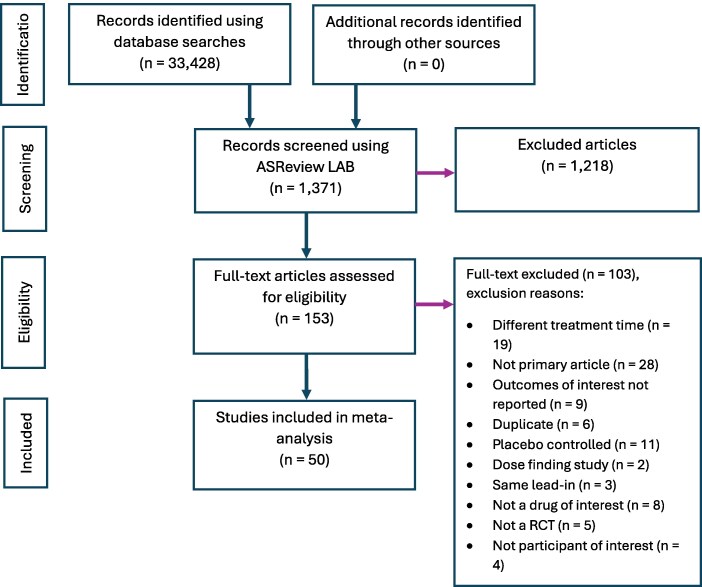
Flow diagram of literature search and reference selection.

A total of 33 428 articles were identified in the first screening (*Figure 1*). We selected articles describing randomized controlled trials on participants with ASCVD which compared different antithrombotic regimens. After reviewing title and abstract of a total of 1371 references, 100 consecutive references were excluded and the review process was stopped, resulting in 153 selected articles for full-text assessment. Ultimately, 50 articles met the inclusion criteria, comprising 60 drug comparisons detailed in the analysis. Characteristics of these trials are summarized in *Table 1*, with additional trial-by-trial specifics provided in Supplementary material online, *Table S1*.

**Table 1:** Summary of characteristics of the included trials

Number of trials	50
Number of drug comparisons	60
Median number of participants	3771 (2362–10 904)
Median follow-up (months)	24.0 (15.0–30.0)
Median age (years)	64.9 (62.1–66.2)
Women	28.0% (23.9–36.0)
Qualifying event	
CAD	50%
Stroke/TIA	32%
PAD	10%
Multiple of the above	8%
Type of drugs used in number of trials^a^	
Platelet aggregation inhibition—platelet aggregation inhibition	41
Platelet aggregation inhibition—vitamin K antagonist	10
Platelet aggregation inhibition—direct oral anticoagulant	6
Vitamin K antagonist (in reference + comparator)	1
DOAC (in reference + comparator)	2
Interventions performed	
PCI	28
CABG	3%
Thrombolysis	7%
PAV intervention/surgery	7%
No intervention	56%

Median reported with 25th and 75th percentile in parentheses. Other reported percentages indicate means.

CAD, coronary artery disease; TIA, transient ischaemic attack; PAD, peripheral arterial disease; DOAC, direct oral anticoagulant; PCI, percutaneous coronary intervention; CABG, coronary artery bypass graft.

^a^See Supplementary material online, *Table S1* for details of the comparisons per trial.

### Clinical outcomes

The 60 drug comparisons encompassed a total of 357 711 patients, followed over a median period of 23.5 months (range 6–60 months). The combined incidence of the composite of ACS and stroke was 21 765 (6.6%), with separate occurrences of ACS and stroke in 13 511 (3.8%) and 9337 (2.8%) respectively. The combined total is lower than the sum of its components, because the composite only included trials in which both outcomes were reported. Major adverse cardiovascular event (composite of ischaemic stroke and ACS plus MALE and/or revascularization rate when reported) was observed in 31 702 (9.4%). Revascularization outcomes, reported in 26 trials, occurred in 9009 (4.3%) of cases. Intracranial haemorrhage occurred in 1793 (0.5%) participants. All-cause death occurred in 19 711 (5.6%) persons, while death due to cardiovascular events comprised 11 168 events (3.3%) and the incidence of death due to bleeding was 1265 (0.4%).

The most commonly used bleeding classification was the TIMI bleeding classification, which was used in 32% of trials. Given that no single bleeding assessment tool was used in 75% or more of trials, intracranial haemorrhage was designated as the primary bleeding outcome. Data on transfusions and hospital admissions due to bleeding complications were only reported in eight and three trials, respectively. Only nine trials reported MALE. Major adverse limb event was therefore solely used in the composite outcome of MACE.

### Meta-regression analysis

**Figure 2:**
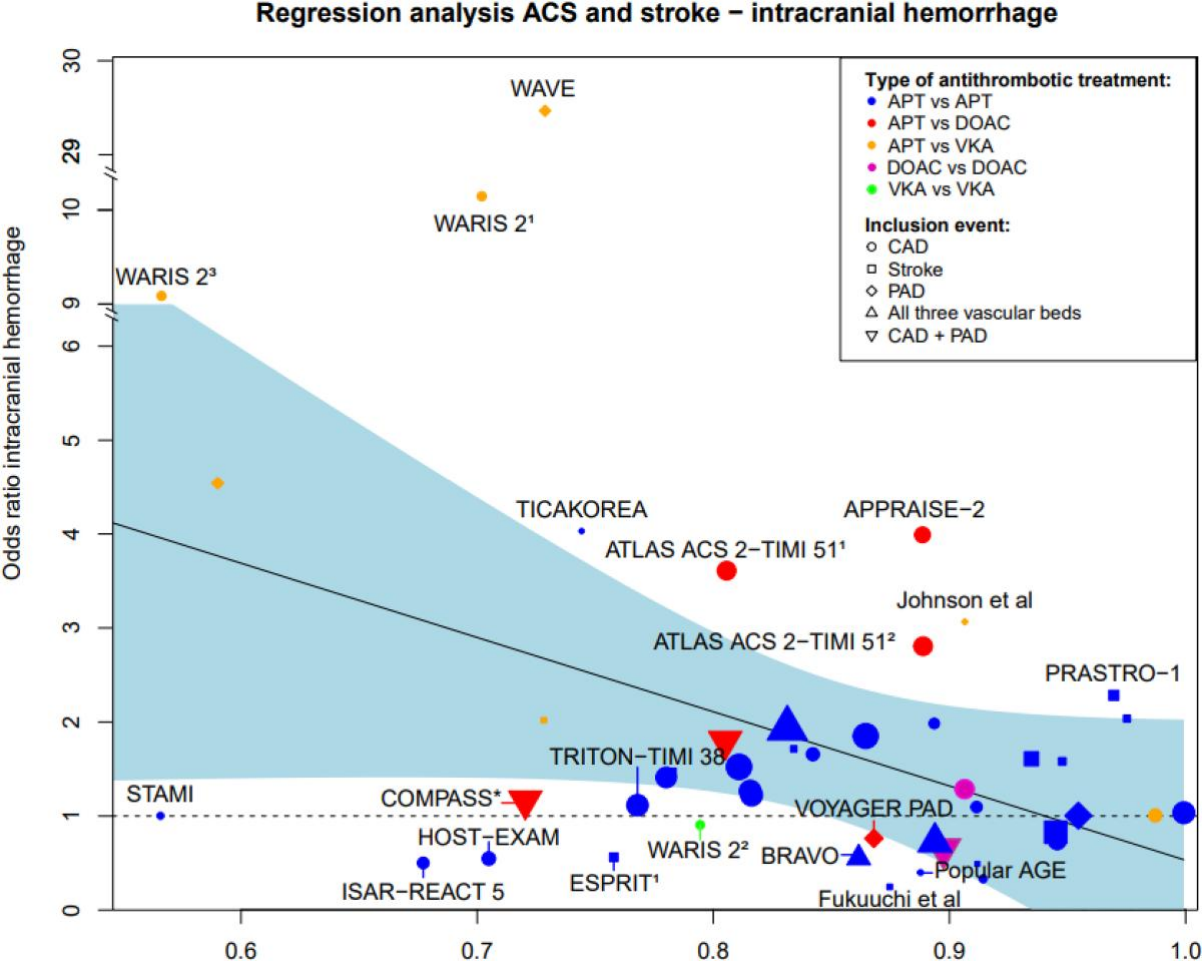
Meta-regression analysis of odds ratio of the combined endpoint of acute coronary syndrome and stroke compared with odds ratio of intracranial bleeding. Specifying comparisons of trials with multiple comparisons: ATLAS ACS 2-TIMI 51^1^ = aspirin + ticlopidine/clopidogrel vs. combination with rivaroxaban 5 mg bid; ATLAS ACS 2-TIMI 51^2^ = aspirin + ticlopidine/clopidogrel vs. combination with rivaroxaban 2.5 mg bid; COMPASS^1^ = aspirin vs. aspirin + rivaroxaban 2.5 mg bid; WARIS^1^ = aspirin 160 mg vs. warfarin INR 2.8–4.2; WARIS 2^2^ = warfarin INR 2.8–4.2 vs. aspirin 75 mg + warfarin INR 2–2.5; WARIS 2^3^ = aspirin 160 mg vs. aspirin 75 mg + warfarin INR 2–2.5; ESPRIT^1^ = aspirin vs. aspirin + dipyridamole 200 mg bid. APT, antiplatelet therapy; DOAC, direct oral anticoagulant; VKA, vitamin K antagonist.

**Figure 3:**
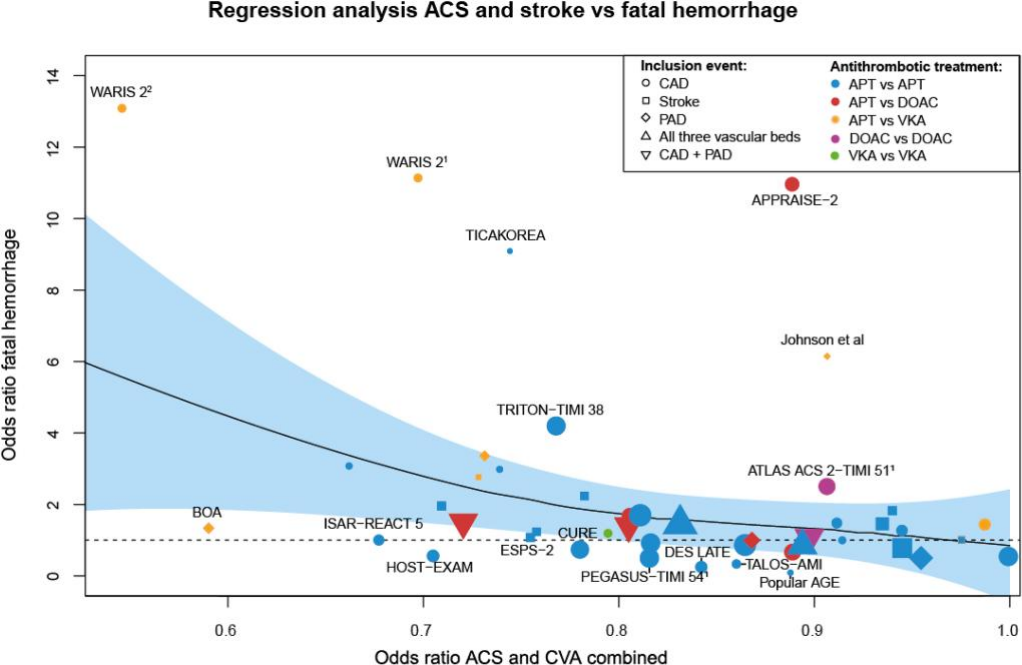
Meta-regression analysis of odds ratio of the combined endpoint of acute coronary syndrome and stroke compared with odds ratio of death due to bleeding. Specifying comparisons of trials with multiple comparisons: ATLAS ACS 2-TIMI 51^1^ = aspirin 75–100 mg + clopidogrel 75 mg qd/ticlopidine 250 mg bid vs. aspirin 75–100 mg + clopidogrel 75 mg qd/ticlopidine 250 mg bid + rivaroxaban 5 mg bid; WARIS 2^1^ = aspirin 160 mg vs. warfarin INR 2.8–4.2; WARIS 2^2^ = warfarin INR 2.8–4.2 vs. aspirin 75 mg + warfarin INR 2–2.5; PEGASUS-TIMI 54^1^ = aspirin 75–150 mg vs. aspirin 75–150 mg + ticagrelor 90 mg qd. APT, antiplatelet therapy; DOAC, direct oral anticoagulant; VKA, vitamin K antagonist.

The odds ratio of the primary composite of ACS and ischaemic stroke (both fatal and non-fatal) was inversely correlated with the odds ratio of intracranial haemorrhage (*Figure 2*). In accordance, a reduction in ACS and ischaemic stroke was also associated with increased odds for fatal haemorrhage (*Figure 3*). A similar inverse relationship was observed between the odds ratio for MACE (comprising ACS, ischaemic stroke, revascularization, and MALE) and intracranial haemorrhage (see Supplementary material online, *Figure S1*) and the relationship between the number of revascularizations and intracranial haemorrhage occurrence (see Supplementary material online, *Figure S2*).

**Figure 4:**
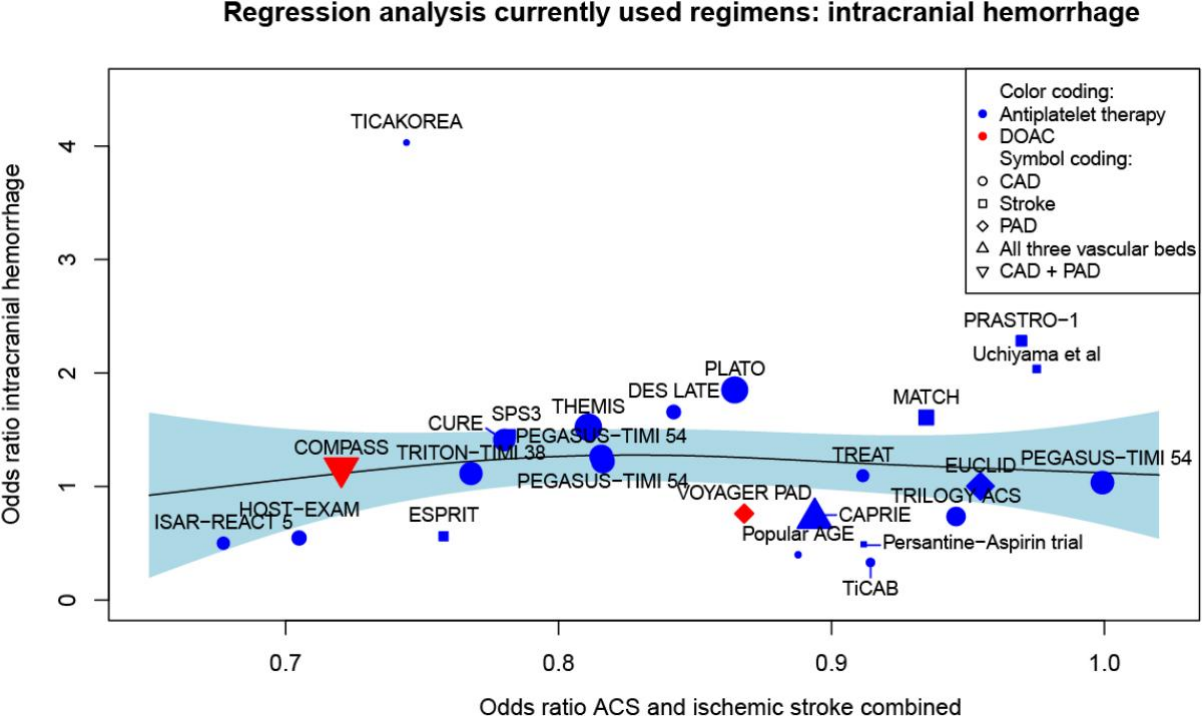
Meta-regression analysis of currently used regimens. Comparison of the odds ratio of the combined endpoint of acute coronary syndrome and stroke compared with odds ratio of intracranial haemorrhage. An interactive version of this figure is available at https://deurvorst.shinyapps.io/Antithromboticregimen/.

**Figure 5:**
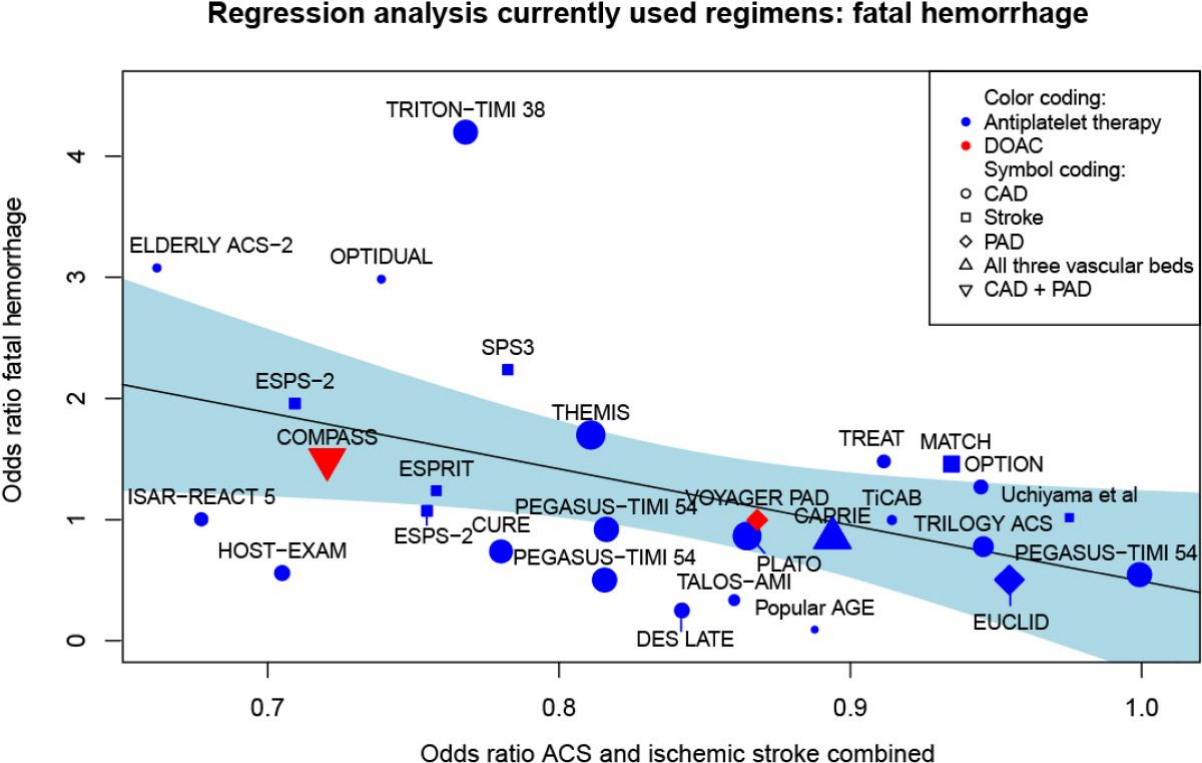
Meta-regression analysis of currently used regimens. Comparison of the odds ratio of the combined endpoint of acute coronary syndrome and stroke compared with odds ratio of death due to bleeding. The TICAKOREA trial is not visible as the odds ratio for a fatal haemorrhage is 9. An interactive version of this figure is available at https://deurvorst.shinyapps.io/Antithromboticregimen/.

Subsequently, (arms of) trials which compared treatment regimens currently not used in clinical practice or recommended in ASCVD guidelines were excluded (*Figures 4* and *5*). The regimens which were excluded compared vitamin K antagonists (VKA),^6–14^ monotherapy with a DOAC,^15^ triple therapy (DAPT + DOAC),^16,17^ vorapaxar,^18^ ticlopidine,^8,17,19–22^ or lotrafiban.^23^ When analysing the remaining trials, the risk of intracranial haemorrhage was attenuated to non-significant. Currently used regimens still show a correlation between ASCVD reduction and an increased risk of fatal haemorrhage, but the slope of risk increase is less steep than in the aggregate analysis. In accordance, the absolute risk of fatal bleeding in trials using only contemporary guideline-recommended regimens is lower than in the aggregate of all trials (0.21 vs. 0.49%)

## Discussion

We performed meta-regression analyses of the antithrombotic efficacy and bleeding risk of antithrombotic regimens. Our primary analysis suggests the expected trade-off between ASCVD reduction and increased bleeding: an increase in antithrombotic driven reduction in ACS and ischaemic stroke risk comes at the cost of an increased risk of intracranial and fatal bleeding. However, when we exclude trials including regimens that are not currently used or recommended, the bleeding costs of ASCVD reduction by intensified antithrombotic treatment are markedly attenuated, with no significant increase in intracranial bleeding and a far more modest remaining increase in fatal bleeding, at very low levels of absolute risk in the included trials.

Combining these observations, it appears that over the years the cardiovascular community has phased out antithrombotic regimens with the most unfavourable cost-benefit profile. A potential caveat in interpreting these data is the patient selection in the included trials. The observed incidence of bleeding events is likely to be underestimated compared with the real-world bleeding risk. Clinical trials exclude patients at a higher risk of bleeding complications (i.e. patients with anaemia, higher age, history of bleeding complications) and often incorporate run-in periods that reduce reported bleeding events. Few of the included trials describe a true run-in period.^8,15^ However, a majority of trials included patients days up to half a year after a cardiovascular event.^6,9–11,17,18,20–40^ Participants experiencing side effects during this phase (i.e. bleeding events) from any anticoagulant therapy were not included in these trials.

When stratifying our outcomes based on the inclusion event (*Figures 2* and *3*; Supplementary material online, *Figure S1* and *S2*), no consistent relationship emerges. In other words, any observed differences in the efficacy and safety of antithrombotic regimens are likely not attributable to the event leading to inclusion.

The association we found of an increased risk of fatal haemorrhage when ASCVD risk is reduced in contemporary regimens seems to be mainly driven by the results of the TRITON-TIMI 38 and the TICAKOREA trial.^41,42^

Apart from the overall association between antithrombotic efficacy and increased bleeding risk, our analysis may also invite a qualitative assessment of which antithrombotic drug comparisons compare particularly favourable or unfavourable in terms of benefit vs. bleeding risk. Formal statistical testing of such comparisons has statistical caveats. The overall picture is that few antithrombotic regimens may appear to stand out positively, but none of such findings were successfully reproduced in subsequent trials.

For future studies on this topic, we aim to provide a benchmark based on our analysis. Therefore, *Figures 3* and *4* have been made available (https://deurvorst.shinyapps.io/Antithromboticregimen/), allowing data from upcoming studies to be entered and compared with currently available data. This tool cannot guide clinical decision-making for individual patients, but it can aid in the interpretation of future trial data. Newly published results can be readily compared with those of preceding trials, particularly in terms of the bleeding costs associated with antithrombotic benefits.

The limitations of our study and the published tool include the reliance on published aggregate analyses without access to individual patient data, which prevents us from addressing exploratory questions regarding subgroups. Individual patient data would also enable the development of better risk classification scores for different patient populations. Secondly, uniform use of bleeding assessment scores would allow to make similar analysis for additional clinical relevant bleeding outcomes.^43^

This analysis includes both historical and contemporary trials with different patient populations. The broad inclusion criteria enhance generalizability of our findings, but the resulting heterogeneity limits the applicability to specific patient populations.

Despite these limitations, we believe that this analysis is helpful in the search for an optimal antithrombotic regime for CVD patients. Existing and future trials comparing antithrombotic regimens will show benefit-to-harm ratios that can be compared with the benchmark we have constructed. This would help substantiate claims for superior benefit-risk ratios, as well as identify disproportionately high bleeding risks for particular antithrombotic regimens.

## Conclusions

In this meta-analysis, we have examined the association between the efficacy and safety of antithrombotic regimens. We found that with currently used antithrombotic regimens, ASCVD event reduction by more intensive treatment no longer increases risk of intracranial bleeding, but this remains the case for fatal haemorrhage. Furthermore, we encourage the application of this benchmark to forthcoming trials. It provides a readily available comparison of new regimens against the established average trade-off between ASCVD prevention and bleeding risk.

## Supplementary Material

qcaf128_Supplementary_Data

## Data Availability

The data underlying this article will be shared on reasonable request to the corresponding author.
