# Supplementary material for: The bleeding costs of intensive vs. less intensive antithrombotic strategies: a meta-regression analysis
Source: Eur Heart J Qual Care Clin Outcomes. 2025 Oct 23;12(5):874–81. doi: 10.1093/ehjqcco/qcaf128 (PMC13458146; doi:10.1093/ehjqcco/qcaf128)
Supplement: Supplementary file 1 [file qcaf128_supplementary_data.docx]

**Supplemental material**

**Figure S1**

*Figure S1. Meta-regression MACE versus intracranial hemorrhage. Trials with missing values on MALE and/or revascularization were not excluded.* *Specifying comparisons of trials with multiple comparisons: ATLAS ACS 2-TIMI 51^1^ = aspirin 75-100mg + clopidogrel 75mg qd/ticlopidine 250mg bid vs aspirin 75-100mg + clopidogrel 75mg qd/ticlopidine 250mg bid + rivaroxaban 5mg bid; WARIS 2^1^ = aspirin 160mg vs warfarin INR 2.8 – 4.2; WARIS 2^2^ = warfarin INR 2.8-4.2 vs aspirin 75mg + warfarin INR 2-2.5; PEGASUS-TIMI 54^1^ = aspirin 75-150mg vs aspirin 75-150mg + ticagrelor 90 mg qd.*
*Abbreviations: APT = antiplatelet therapy; DOAC = direct oral anticoagulant; VKA = vitamin K antagonist*

**Figure S2**

*Figure S2. Meta-regression analysis revascularization rate versus intracranial hemorrhage. Same inverse relation is observed compared to ACS and stroke versus intracranial hemorrhage even though less studies had revascularization data available. Specifying comparisons of trials with multiple comparisons: ATLAS ACS 2-TIMI 51^1^ = aspirin 75-100mg + clopidogrel 75mg qd/ticlopidine 250mg bid vs aspirin 75-100mg + clopidogrel 75mg qd/ticlopidine 250mg bid + rivaroxaban 5mg bid; WARIS 2^1^ = aspirin 160mg vs warfarin INR 2.8 – 4.2; WARIS 2^2^ = warfarin INR 2.8-4.2 vs aspirin 75mg + warfarin INR 2-2.5; WARIS 2^3^ = aspirin 160mg vs aspirin 75mg + warfarin INR 2-2.5*
*Abbreviations: APT = antiplatelet therapy; DOAC = direct oral anticoagulant; VKA = vitamin K antagonist*

**Search strategy PubMed**

| **9** | (#5 AND #6 AND #7) NOT (#8)) |
| --- | --- |
| **8** | ("Animals"[Mesh] NOT "Humans"[Mesh]) |
| **7** | (randomized controlled trial[pt] OR controlled clinical trial[pt] OR randomized[tiab] OR placebo[tiab] OR clinical trials as topic[mesh:noexp] OR randomly[tiab] OR trial[ti] NOT (animals[mh] NOT humans [mh])) |
| **6** | ("Aspirin"[Mesh]) OR ("Platelet Aggregation Inhibitors"[Mesh]) OR ("platelet inhibitor*"[TIAB]) OR ("Antiaggregant*"[TIAB]) OR ("Anti-aggregant*"[TIAB]) OR ("aggregation inhib*"[TIAB]) OR ("antiplatelet"[TIAB]) OR ("acetylsalicylic acid"[TIAB]) OR ("Xarelto"[TIAB]) OR ("Rivaroxaban"[TIAB]) OR ("Dipyridamole"[Mesh]) OR ("persantin"[TIAB]) OR ("Clopidogrel"[Mesh]) OR (“Plavix”[TIAB]) OR (“iscover”[TIAB]) OR ("Ticlopidine"[Mesh]) OR (“Ticlopidine”[TIAB]) OR ("Ticagrelor"[Mesh]) OR ("Brilique"[TIAB]) OR ("Prasugrel Hydrochloride"[Mesh]) OR ("efient"[TIAB]) OR (“Cangrelor”[TIAB]) OR (“Kengrexal”[TIAB]) OR ("4-Hydroxycoumarins"[Mesh]) OR ("Coumarins"[TIAB]) OR ("VKA"[TIAB]) OR ("vitamin K antagonist*"[TIAB]) OR ("warfarin"[TIAB]) OR ("acenocoumaro*"[TIAB]) OR ("fenprocoumo*"[TIAB]) OR ("DOAC"[TIAB]) OR ("apixaban"[TIAB]) OR ("eliquis"[TIAB]) OR ("edoxaban"[TIAB]) OR ("lixiana"[TIAB]) OR ("dabigatran"[TIAB]) OR ("pradaxa"[TIAB]) OR ("rivaroxaban"[MESH]) OR ("edoxaban" [Supplementary Concept]) OR ("Dabigatran"[Mesh]) OR ("apixaban" [Supplementary Concept]) |
| **5** | #1 OR #2 OR #3 OR #4 |
| **4** | ("Arteriosclerosis"[Mesh]) OR (“Atherosclero*”[TIAB]) OR (“Arteriolosclero*”[TIAB]) OR (“atherothrombotic event*”[TIAB]) OR ("Arterial Occlusive Diseases"[Mesh]) |
| **3** | ("Peripheral Arterial Disease"[Mesh]) OR (‘limb isch*’[TIAB]) OR (“peripheral arterial disease*”[TIAB]) OR (“claudicatio*”[TIAB]) OR (“PAD”[TIAB]) |
| **2** | ("Brain Ischemia"[Mesh]) OR ("Carotid Artery Diseases"[Mesh]) OR ("Stroke, Lacunar"[Mesh]) OR ("Intracranial Arterial Diseases"[Mesh]) OR ("Intracranial Embolism and Thrombosis"[Mesh]) OR ("Stroke"[Mesh]) OR ("CEREBROVASCULAR disease*"[tiab]) OR ("CVA"[TIAB]) OR ("TIA"[TIAB]) OR ("transient ischemic attack*"[TIAB]) OR ("cerebrovascular event*"[TIAB]) OR ("cerebrovascular accident*"[TIAB]) OR (“brain isch*”[TIAB]) OR (“brain hypox*”[TIAB]) OR (“carotid stenos*”[TIAB]) OR (“carotid artery narrow*”[TIAB]) OR (“brain infarct*”[tiab]) |
| **1** | ("Myocardial Ischemia"[Mesh]) OR ("Myocardial infarction*"[TIAB]) OR ("STEMI"[TIAB]) OR ("NSTEMI"[TIAB]) OR (“N-STEMI”[TIAB]) OR (“non-STEMI”[TIAB]) OR ("heart attack"[TIAB]) OR ("Myocardial isch*"[TIAB]) OR (“angin*”[TIAB]) OR (“coronary artery diseas*”[TIAB]) OR (“CAD”[TIAB]) OR (“ischemic heart diseas*”[TIAB]) OR (“acute coronary syndrom*”[TIAB]) OR (“ACS”[TIAB]) OR (“coronary occlusion*”[TIAB]) OR (“coronary stenosis”[TIAB]) OR ("Coronary Thrombosis"[Mesh]) OR (“instent thrombos*”[TIAB]) OR (“in-stent thrombos*”[TIAB]) |

| Trial name | Year | Arms | N | Reference therapy | Experimental therapy | Index event | Additional inclusion criteria | Intervention |
| --- | --- | --- | --- | --- | --- | --- | --- | --- |
| ATLAS ACS 2-TIMI 51(23) | 2012 | 3 | 15526 | Aspirin 75-100 mg/day + clopidogrel 75 mg/ticlopidine 250 mg b.i.d. | Aspirin 75-100 mg/day + clopidogrel 75 mg/ticlopidine 250mg b.i.d. + rivaroxaban 2x5 mg; same dose aspirin + clopidogrel/ticlopidine with rivaroxaban 2,5 mg b.i.d. | (N)STEMI or unstable AP | If <55 years old additional DM or previous MI | PCI/CABG: 60.4%  No intervention: 39.6% |
| APPRAISE-2(35) | 2011 | 2 | 7392 | Aspirin <325 mg/day + P2Y12-receptor antagonist | Aspirin <325 mg/day + P2Y12-receptor antagonist + apixaban 5 mg b.i.d. | (N)STEMI or unstable AP | Two or more additional risk factors: >65 years old; DM, previous MI last 5 years, EF <40%, eGFR <60, no revascularization at index event | PCI: 44%; CABG: 0.6%; no intervention 55.4% |
| BOA(39) | 2000 | 2 | 2650 | Aspirin 80 mg/day | Phenprocoumon/acenocoumarol INR: 3.0-4.5 | Bypass surgery leg |  | Bypass surgery: 100% |
| BRAVO(32) | 2003 | 2 | 9190 | Aspirin 75-325 mg/day | Aspirin 75-325 mg/day + lotrafiban 30/50 mg b.i.d. | MI or UAP; ischemic stroke; TIA; “double bed” vascular disease |  | NA |
| CHAMP(12) | 2002 | 2 | 5059 | Aspirin 162 mg/day | Aspirin 81 mg/day + warfarin INR: 1.5-2.5 | MI |  | Thrombolysis: 30.5%; 69.5% conservative |
| COMPASS(7) | 2017 | 3 | 27395 | Aspirin 100 mg/day | Rivaroxaban 5mg b.i.d.; Aspirin 100 mg/day and Rivaroxaban 2,5 mg b.i.d. | “Stable” CAD or PAD | If <65 years old required to have atherosclerosis of two vascular beds or two additional risk factors: smoking, DM, eGFR <60, HF or stroke |  |
| CURE(40) | 2001 | 2 | 12562 | Aspirin 75-325 mg/day | Aspirin 75-325 mg/day + Clopidogrel 75 mg q.d. | NSTEMI |  | PCI: 21.2%; 16.5% CABG, 1.6% thrombolysis; 60.7% no intervention |
| CAPRIE(41) | 1996 | 2 | 19185 | Aspirin 325 mg/day | Clopidogrel 75 mg q.d. | ACS; CVA or PAD |  | Thrombolysis not specified, otherwise no intervention performed |
| DESLATE(42) | 2014 | 2 | 5045 | Aspirin 100-200 mg/day | Aspirin 100-200 mg/day + clopidogrel 75 mg q.d. | 12 months post PCI | No MACE or major bleeding preceding 12 months |  |

| Trial name | Year | Arms | N | Reference therapy | Experimental therapy | Index event | Additional inclusion criteria | Intervention |
| --- | --- | --- | --- | --- | --- | --- | --- | --- |
| EUCLID(19) | 2017 | 2 | 13885 | Clopidogrel 75 mg q.d. | Ticagrelor 90 mg b.i.d. | PAD |  | Any leg revascularization: 56.7%; no intervention: 43.3% |
| ELDERLY ACS-2(43) | 2018 | 2 | 1443 | Aspirin 75-100 mg/day + clopidogrel 75 mg q.d. | Aspirin 75-100mg/day + prasugrel 5 mg q.d. | ACS | >74 years old | No intervention: 100% |
| ESPS-2(44) | 1996 | 3 | 4953 | Dipyridamole 200 mg b.i.d. | Aspirin 50 mg/day | CVA or TIA | >18 years old | No intervention: 100% |
|  |  |  |  | Aspirin 50 mg/day | Aspirin 50mg/day + dipyridamole 200mg b.i.d. |  |  |  |
|  |  |  |  | Dipyridamole 200 mg b.i.d. | Aspirin 50mg/day+ dipyridamole 200 mg b.i.d. |  |  |  |
| ESPRIT*(14) | 2006 | 2* | 2739 | Aspirin 30-325 mg/day | Aspirin 30-325 mg/day + dipyridamole 200 mg b.i.d. | TIA or minor stroke |  | No intervention: 100% |
| ESPRIT*(15) | 2007 | 2* | 1068 | Aspirin 30-325 mg/day | Phenprocoumon (or acenocoumarol or warfarin) INR: 2-3 | TIA or minor stroke |  | No intervention: 100% |
| HOST-EXAM(21) | 2021 | 2 | 5438 | Aspirin 100 mg/day | Clopidogrel 75 mg q.d. | 12 months after PCI | No MACE or major bleeding preceding 12 months |  |
| ISAR-REACT 5(45) | 2019 | 2 | 4018 | Aspirin <100mg/day + ticagrelor 90 mg b.i.d. | Aspirin <100mg/day + prasugrel 10 mg q.d. | (N)STEMI or UAP |  | PCI: 84.2%; CABG: 2%; no intervention: 13.8% |
| LoWASA(18) | 2004 | 2 | 3300 | Aspirin 75 mg/day | Aspirin 75 mg/day + warfarin 1.25 mg | MI |  | PCI: 4%; CABG: 1%; thrombolysis 41!; no intervention: 54% |
| MATCH(46) | 2004 | 2 | 7599 | Clopidogrel 75 mg q.d. | Aspirin 75 mg/day + clopidogrel 75 mg q.d. | TIA or stroke with an additional risk factor | Additional risk factor: previous ischaemic stroke or MI, AP, DM, or PAD – within previous 3 years | No intervention: 100% |
| OPTION(34) | 2022 | 2 | 4451 | Aspirin 100 mg/day + clopidogrel 75 mg q.d. | Indobufen 100 mg b.i.d. + clopidogrel 75 mg q.d. | Stable CAD or UAP | 18-75 years old | PCI: 100% |
| OPTIDUAL(17) | 2016 | 2 | 1385 | Aspirin 75-160 mg/day | Aspirin 75-160 mg/day + clopidogrel 75 mg q.d. | 12 months after PCI | ≥1 lesion with stenosis >50% located in a native vessel ≥2.25 mm in diameter | PCI: 100% |
| PLATO(47) | 2009 | 2 | 18624 | Clopidogrel 75 mg q.d. | Ticagrelor 90 mg b.i.d. | (N)STEMI |  | PCI: 61%; CABG: 4.5%; no intervention 34.5% |

| Trial name | Year | Arms | N | Reference therapy | Experimental therapy | Index event | Additional inclusion criteria | Intervention |
| --- | --- | --- | --- | --- | --- | --- | --- | --- |
| PEGASUS-TIMI 54(10) | 2015 | 3 | 21162 | Aspirin 75-150 mg/day | Aspirin 75-150mg/day + ticagrelor 90 mg b.i.d. | 1-3 year after (N)STEMI or UAP; | >50 years and an additional risk factor: >65 years, DM, previous MI, multivessel CAD, eGFR <60 | PCI 83%; no intervention 17% |
|  |  |  |  | Aspirin 75-150 mg/day | Aspirin 75-150 mg/day + ticagrelor 60 mg b.i.d. |  |  |  |
|  |  |  |  | Aspirin 75-150 mg/day + ticagrelor 60 mg b.i.d. | Aspirin 75-150 mg/day + ticagrelor 90 mg b.i.d. |  |  |  |
| PRASTRO-1(26) | 2019 | 2 | 3743 | Prasugrel 3,75 mg q.d. | Clopidogrel 75 mg q.d. | Ischemic stroke | 20-74 years old | No intervention: 100% |
| Popular AGE(13) | 2020 | 2 | 1002 | Clopidogrel 75mg q.d. on top of 86% Aspirin, 16% oral anticoagulation | Ticagrelor 90 mg b.i.d. , 86% Aspirin, 20% oral anticoagulation, | (N)STEMI | >70 years old | PCI 47%; CABG: 16.5%; no intervention 36.5% |
| PERFORM(11) | 2011 | 2 | 19100 | Terutroban 30 mg q.d. | Aspirin 100 mg/day | Stroke or TIA |  | No intervention: 100% |
| PRoFESS(29) | 2008 | 2 | 20332 | Aspirin 25 mg/day + dipyramidol 200 mg b.i.d. | Clopidogrel 75 mg q.d. | Stroke | >55 years old | No intervention: 100% |
| REAL-LATE + ZEST-LATE(27) | 2010 | 2 | 2701 | Aspirin 100-200 mg/day | Aspirin 100-200 mg/day + clopidogrel 75 mg q.d. | ACS + SAP |  | PCI: 100% |
| S-ACCESS(31) | 2008 | 2 | 1499 | Sarpogrelate 100 mg b.i.d. | Aspirin 81 mg/day | CVA | >20 years old | No intervention: 100% |
| STAMI(30) | 2001 | 2 | 1470 | Aspirin 160 mg/day | Ticlopidine 250 mg b.i.d. | MI | >18 years old | Thrombolysis: 100% |
| SPIRIT(8) | 2004 | 2 | 1316 | Aspirin 30-100 mg/day | Phenprocoumon/acenocoumerol/warfarin INR: 3-4,5 | TIA or minor stroke |  | No intervention: 100% |
| SPS3(9) | 2012 | 2 | 3020 | Aspirin 325 mg/day | Aspirin 325 mg/day+ clopidogrel 75 mg | Stroke | >30 years old | No intervention: 100% |
| TRILOGY ACS(28) | 2012 | 2 | 9326 | Aspirin ≤100 mg/day + clopidogrel 75 mg | Aspirin ≤100 mg/day + prasugrel 5-10 mg q.d. | NSTEMI or UAP with additional risk factor | Risk factor: >60 years old; DM, previous MI or coronary revascularization | No intervention: 100% |
| TICAKOREA(48) | 2019 | 2 | 800 | Aspirin 100 mg/day + clopidogrel 75 mg q.d. | Aspirin 100 mg/day + ticagrelor 90 mg b.i.d. | (N)STEMI | >18 years old | PCI: 83.5%; CABG: 2.1%; no intervention: 14.4% |
| TiCAB(49) | 2019 | 2 | 1859 | Aspirin 100 mg/day | Ticagrelor 90 mg b.i.d. | UAP, SAP or NSTEMI | Planned CABG | CABG: 100% |
| TREAT(50) | 2019 | 2 | 3799 | Aspirin 75-100 mg/day + clopidogrel 75 mg q.d. | Aspirin 75-100mg/day + ticagrelor 90 mg b.i.d. | STEMI | <75 years old | Thrombolysis 100%, after which 57% underwent PCI |
| THEMIS(51) | 2019 | 2 | 19220 | Aspirin 75-150 mg/day | Aspirin 75-150mg/day + ticagrelor 60 mg b.i.d. | Stable CAD and diabetes mellitus | >50 years old | No intervention: 100% |
| TRITON-TIMI38(52) | 2007 | 2 | 13608 | Aspirin 75-162 mg/day + clopidogrel 75 mg q.d. | Aspirin 75-162mg/day + prasugrel 10 mg q.d. | ACS |  | PCI: 99%; CABG: 1% |
| Trial name | **Year** | **Arms** | **N** | **Reference therapy** | **Experimental therapy** | **Index event** | **Additional inclusion criteria** | **Intervention** |
| TWILIGHT(24) | 2019 | 2 | 7119 | Ticagrelor 90mg b.i.d. | Aspirin 80-100mg/day + ticagrelor 90 mg b.i.d. | NSTEMI, stable CAD, UAP and additional risk factor | Clinical: ≥65 years, female, troponin positive, DM, CKD. Angiographic: multivessel CAD, length >30 mm, thrombotic lesion, bifurcation lesion with two stents, obstructive LM or proximal LAD or atherectomy for calcified lesion | PCI 100% |
| TALOS-AMI(20) | 2021 | 2 | 2697 | Aspirin 100 mg/day + clopidogrel 75 mg q.d. | Aspirin 100 mg/day + ticagrelor 90 mg b.i.d. | MI |  | PCI: 100% |
| TASS(16) | 1989 | 2 | 3069 | Aspirin 1300 mg/day | Ticlopidine 250 mg b.i.d. | Stroke or TIA |  | No intervention: 100% |
| TACIP(22) | 2003 | 2 | 2113 | Triflusal 600 mg/day | Aspirin 325 mg/day | Stroke or TIA | ≥40 years old | No intervention: 100% |
| TRA 2P-TIMI 50(25) | 2012 | 2 | 26449 | Aspirin +/- Thienopyridine | Aspirin +/- Thienopyridine and Vorapaxar 2,5 mg q.d. | MI; stroke; PAD |  | NA; exclusion if ‘planned intervention’ |
| VOYAGER PAD(53) | 2020 | 2 | 6564 | Aspirin 100 mg/day | Aspirin 100 mg/day + rivaroxaban 2,5mg b.i.d. | Revascularization leg | >50 years old | Revascularization endovascular: 65.4%; surgical: 34.6% |
| WARSS(54) | 2002 | 2 | 2206 | Aspirin 325 mg/day | Warfarin INR: 1,4 - 2,8 | Ischemic stroke |  | No intervention: 100% |
| WARIS 2(36) | 2002 | 3 | 3630 | Aspirin 160 mg/day | Warfarin INR 2.8 - 4.2 | MI | <75 years old | Thrombolysis: 54% |
|  |  |  |  | Warfarin INR 2.8 - 4.2 | Aspirin 75 mg + warfarin INR: 2 - 2,5 |  |  |  |
|  |  |  |  | Aspirin 160 mg/day | Aspirin 75 mg + warfarin INR: 2 - 2,5 |  |  |  |
| WAVE(6) | 2007 | 2 | 2206 | Aspirin81-325mg/day or/and ticlopidine or clopidogrel | Aspirin81-325mg/day or/and ticlopidine/clopidogrel + warfarin/acenocoumarol INR 2-3 | PAD | 35-85 years old | Both intervention and stable disease |
| Persantin-Aspirin trial(55) | 1985 | 2 | 890 | Aspirin 1300 mg/day | Aspirin 1300 mg/day + persantin 75 mg q.i.d. | Carotid territory TIA |  | No intervention: 100% |
| Uchiyama et al(33) | 2009 | 2 | 1862 | Clopidogrel 75 mg q.d. | Ticlopidine 200 mg q.d. | Ischemic stroke | 20-80 years old | No intervention: 100% |
| Johnson et al(37) | 2004 | 2 | 831 | Aspirin 325 mg/day | Aspirin 325 mg/day + warfarin INR 1.4 - 2.8 | Elective surgery for PAD |  | Surgical revascularization: 100% |
| Fukuuchi et al(56) | 2008 | 2 | 1151 | Clopidogrel 75 mg q.d. | Ticlopidine 200 mg q.d. | Ischemic stroke | 20-80 years old | No intervention: 100% |

*Table S1. Summary of included trials. Abbreviations: NSTEMI: Non-ST-elevated myocardial infarction; STEMI: ST-elevated myocardial infarction; ACS: acute coronary syndrome; MI: myocardial infarction; TIA: transient ischemic accident; PCI: percutaneous coronary intervention; CABG: coronary artery bypass graft; SAP: stable angina pectoris; UAP: unstable angina pectoris q.d.: quaque die, once a day; b.i.d.: bis in die, twice a day; q.i.d.: quarter in die = four times a day; LM: Left Main coronary artery; LAD: Left Anterior Descending artery*
